# Analysis of RV components after reoperation of the right ventricular outflow tract in patients with Tetralogy of Fallot

**DOI:** 10.1186/1532-429X-17-S1-Q91

**Published:** 2015-02-03

**Authors:** Abdullah M AL Bulushi, Felicitas Frettlöh, Dominik Daniel Gabbert, Ines Kristo, Andreas Entenmann, Christopher Hart, Inga Voges, Minh H Pham, Michael Jerosch-Herold, Philip Wegner, Ana C Andrade, Hans-Heiner Kramer, Carsten Rickers

**Affiliations:** 1 Congenital Heart Disease, Pediatric Cardiology, University Hospital Schleswig Holstein, Kiel, Germany, Kiel, Germany; 2 Department of Radiology, Harvard Medical School,Women's Hospital Boston, Boston, MA, USA

## Background

In patients with repaired Tetralogy of Fallot (TOF), reoperation of the right ventricular outflow tract (RVOT) due to pulmonary regurgitation and volume overload is often necessary. This study aims to analyse the functional recovery of the right ventricle (RV) and the RVOT after a reoperation using volumetrical data from cine MRI.

## Methods

31 patients (20.6 ± 10.3 years) underwent an MRI examination before and after (20 ± 18 months) pulmonary valve replacement or reconstruction (PVR) for the volume overload of the RV. For segmental analysis of the RV, a custom made analysis software was developed. The method provides an automatic contour detection algorithm for the determination of the RV blood volume and enables the user to segregate the blood volume of the RVOT and the corpus based on anatomic landmarks and to generate volume-time-curves over a cardiac cycle. The analysis of volume-time-curves of the RV allowed calculation of Peak-Filling-Rate (PFR) and Time-to-Peak-Filling-Rate (TPFR), which are surrogate parameters for diastolic dysfunction.

## Results

A significant reduction in blood volumes of the total RV was present after a PVR (RV-ESV -18.4 ±23.6 ml/m^2^, p=0.018, RV-EDV -28.7 ±26.3 ml/m^2^, p<0.01). Pulmonary insufficiency was also significantly reduced (-48.7 ±48.9%, p<0.001). In a subgroup of 22 pts the RVOT was separately analysed and showed a significant reduction after PVR (RVOT-ESV -5.5 ±6.1 ml/m^2^, p<0.001, RVOT-EDV -7.3 ±7.9 ml/m^2^, p<0.001). The PFR decreased (-149.2 ±261.8 ml/s, p=0.001) and the TPFR increased (0.06 ±0.12s, p=0.041), indicating improvement of RV diastolic function. Additionally, late gadolinium imaging showed that non-viable tissue (patch material and scar tissue) was present in 5.7 ±3.8% of the RV mass and in 11.9 ±7.2% of the RVOT mass before operation and did not change on follow-up.

## Conclusions

After reoperation of the RVOT in TOF patients the systolic and diastolic function of the right ventricular components (RVOT and corpus) improved within less than a year. The end-diastolic and end-systolic blood volumes decreased significantly in the RVOT despite its burden of the non-viable tissue and the total RV. The dedicated analysis software enables the user to assess the components of the RV in a time-efficient and precise manner.

## Funding

N/A.

